# Impact of the COVID-19 pandemic waves on adults with moderate-to-severe atopic dermatitis in the Dutch general population: a population-based longitudinal cohort study

**DOI:** 10.3389/fpubh.2025.1641763

**Published:** 2025-12-02

**Authors:** Rui Chen, Laura Loman, Douwe Postmus, Marie L. A. Schuttelaar

**Affiliations:** 1 Department of Dermatology, University Medical Center Groningen, University of Groningen, Groningen, Netherlands; 2 Department of Epidemiology, University Medical Center Groningen, University of Groningen, Groningen, Netherlands

**Keywords:** atopic dermatitis, COVID-19, longitudinal study, mental health, wellbeing, social functioning, loneliness

## Abstract

**Background:**

This study aimed to investigate the impact of COVID-19 waves on mental health, pandemic-related wellbeing, and social functioning in adults with moderate-to-severe atopic dermatitis (AD) and without AD in the Dutch general population.

**Methods:**

Between 2020 and 2022, 31 COVID-19 questionnaires (COVQs) were sent to 140,145 Lifelines adult participants, collecting data on mental health [major depression disorder (MDD), generalized anxiety disorder (GAD)], pandemic-related wellbeing [COVID-19-related concerns, quality of life (QoL)], and social functioning (loneliness, social relations). AD information was collected through a digital questionnaire sent to all Lifelines adults in 2020. Data were divided into three waves according to the COVID-19 timeline in the Netherlands: March–June 2020 (wave 1); July 2020–June 2021 (wave 2); July 2021–October 2022 (wave 3). Generalized linear mixed models were performed for each outcome.

**Results:**

In total, 49,216 participants with 790,936 completed COVQs were included. Compared with wave 1, almost all health-related outcomes, including the prevalence of MDD and GAD, COVID-19-related concerns, loneliness, and QoL, had returned to similar levels by wave 3 in the overall population, while social relations remained impaired. Furthermore, participants with moderate-to-severe AD, women, young adults (18–29 years), and those with a history of mental health problems showed worse health-related outcomes than their counterparts.

**Conclusion:**

Both participants with and without moderate-to-severe AD showed partial recovery in mental health and wellbeing by the third COVID-19 wave; however, social relations remained impaired. Individual with moderate-to-severe AD, women, younger adults, and those with pre-existing mental health problems were more affected overall. Future studies should focus on developing strategies to improve social connectedness, and vulnerable groups warrant continued attention.

## 1 Introduction

Due to the COVID-19 pandemic, governments worldwide implemented measures such as lockdowns, curfews, remote work mandates, and social distancing to curb the spread of the virus, drastically altering people's lives (1). The impact of COVID-19 on diverse populations has been extensively investigated (2, 3).

AD is a common chronic inflammatory skin disease, has significant impact on patients' quality of life (QoL) (4). Previous studies indicated that individuals with AD are more likely to develop anxiety, depression, and even suicide attempts (5). Additionally, a US population-based study showed that 51.3% of AD adults reported restricted lifestyle, leading to 39.1% avoided social interactions (6). Moreover, these negative effects often aggravated with the severity of AD (6, 7). Conclusions from previous cross-sectional studies indicated that AD patients suffered more stress, anxiety, and worries than patients without AD during the COVID-19 (8–10). However, evidence from longitudinal studies is lacking. Moreover, comparative studies about the COVID-19 impacts on adults with and without AD are limited (9, 11).

As the COVID-19 pandemic has gone through several waves in the Netherlands, the lockdown and quarantine requirements have changed in parallel. After the first COVID-19 case was confirmed in February 2020, “intelligent lockdown,” including avoiding social contact, working remotely, and closing public places was implemented in March and gradually eased in June (1). However, with the increased new cases from July, nationwide partial and hard lockdown were conducted in October and December 2020, respectively (1). In July 2021, the number of COVID-19 cases increased rapidly due to the Delta variant, and then with the Omicron variant detected in November 2021, strict measures and a hard lockdown were announced again, with some relief from January 2022, such as schools reopening, and shops reopening with conditions (1). Data on the speed of daily new confirmed cases and reproduction numbers over time are consistent with measurements implemented by the Dutch government (12, 13).

Few studies have compared differences among populations across multiple COVID-19 waves. Longitudinal studies from the UK suggested that repeated waves may lead to sustained declines in population mental wellbeing (14, 15). However, evidence on long-term patterns of mental health, wellbeing, and social functioning after the second wave remains limited, particularly among adults with moderate-to-severe AD in the Dutch general population. Although the COVID-19 pandemic is no longer a global health emergency, its psychological and social effects may persist (16). Therefore, this study aims to investigate the impact of repeated COVID-19 waves on mental health, pandemic-related wellbeing, and social functioning of individuals with moderate-to-severe AD and those without AD in the Dutch general population.

## 2 Materials and methods

### 2.1 Subjects

The current study is conducted within the Lifelines COVID-19 cohort study (17). Lifelines is a multi-disciplinary prospective population-based cohort study examining in a unique three-generation design the health and health-related behaviors of 167,729 persons living in the North of The Netherlands (18). It employs a broad range of investigative procedures in assessing the biomedical, social-demographic, behavioral, physical and psychological factors which contribute to the health and disease of the general population, with a special focus on multi-morbidity and complex genetics (18).

The Lifelines COVID-19 project is an ongoing project aimed at broadly investigate the impact of COVID-19 pandemic on the Dutch population (17). The current study includes a total of 31 COVID-19 questionnaires (COVQs) that were distributed continuously from March 2020 to October 2022. The first COVID-19 questionnaire (COVQ) was sent out on March 30, 2020 to 140,145 adult Lifelines participants with known email address (17). The first six questionnaires (Q1–Q6) were sent out weekly, and questionnaires from Q7 were distributed bi-weekly or monthly. Participants who did not complete any of the first seven questionnaires did not receive any follow-up COVQ. Additionally, participants who had completed at least two previous COVQs (~65,500 participants) were invited to participate in the 18th and subsequent COVQs.

Information related to AD was collected by sending out a digital add-on skin questionnaire (SKIQ) to the adult Lifelines participants (*N* = 135,950), between February and May 2020 (19).

The study was conducted in accordance with the Declaration of Helsinki, and approved by the Medical Ethics Committee of the University Medical Center Groningen (reference number 2007/152 and 2019/571).

### 2.2 Definitions

#### 2.2.1 Participants with AD

Participants with AD were identified based on self-reported physician-diagnosed AD in lifetime (Supplementary Figure S1).

Participants' self-reported AD severity was assessed using the Patient-Oriented Eczema Measure (POEM; 0–28), with total scores of 0–7 classified as mild AD and 8–28 as moderate-to-severe AD (20).

#### 2.2.2 COVID-19 waves

All 31 COVQs conducted at different time points were grouped into three waves based on the COVID-19 timeline, the growth rate of daily confirmed cases, and the reproduction number in the Netherlands (1, 12, 13). Wave 1 (W1): March 2020—June 2020 (COVQ 1–9), wave 2 (W2): July 2020—June 2021 (COVQ 10–21), wave 3 (W3): July 2021—October 2022 (COVQ 22–29).

### 2.3 Socio-demographic variables

Age was calculated as 2020 (the year first COVQ was sent) minus the participant's year of birth. Employment status referred to the participants' situation when completing the first COVQ.

Education level was derived from the most recent available data and categorized into three levels according to the Lifelines standard: low (no education, primary education, lower or preparatory secondary vocational education, junior general secondary education), intermediate (secondary vocational education or work-based learning pathway, senior general secondary education, pre-university secondary education), and high (higher vocational education, university education) (21).

Income level was obtained from the COVQ questionnaire, which asked: “What was your personal net income before the corona crisis?” Twelve response options were provided, ranging from “lower than €500” to “above €7500.” These were categorized into three levels based on quartiles: low ( ≤ €1000), intermediate (€1001–€3000), and high (> €3000).

Moreover, a history of mental health problems was defined as having a diagnosis of major depressive disorder (MDD) or generalized anxiety disorder (GAD) based on the most recent Mini-International Neuropsychiatric Interview (M.I.N.I.) data from the first two Lifelines assessments (2007–2015 and 2014–2019). Participants diagnosed with either MDD or GAD in these assessments were classified as having a history of mental health problems.

Data on COVID-19 vaccination status were first collected during COVQ 22 and followed up until COVQ 29. Participants were asked, “Are you vaccinated against the corona virus?” and their responses were categorized as “yes” or “no.”

### 2.4 Outcome measures

#### 2.4.1 Mental health

Mental health status included current MDD and GAD, with “current” referring to the diagnosis made based on participants' responses to COVQs. Symptoms of MDD and GAD were collected using the M.I.N.I., which has been used in earlier studies (3), and it is compatible with international diagnostic criteria like DSM-IV and ICD-10 (22) (see Supplementary Tables S1, S2 for overview and detailed items in COVQs, as well as the following outcomes).

#### 2.4.2 Pandemic-related wellbeing

Pandemic-related wellbeing was assessed by COVID-19-related concerns and QoL. COVID-19-related concerns were assessed using a non-validated question specifically designed for the Lifelines COVID-19 cohort study. Participants were asked to rate their level of concerns regarding COVID-19, with responses measured on a scale from 1 (not worried) to 10 (extremely worried). QoL was assessed by asking participants to rate their QoL on a scale from 1 to 10, with higher scores indicating better QoL (23).

#### 2.4.3 Social functioning

##### 2.4.3.1 Loneliness

The level of loneliness was measured by using the validated three-item UCLA loneliness scale (University of California, Los Angeles) (24). This scale assessed the frequency of feeling a lack of companionship, feeling left out, and feeling isolated from others, with each item scored from 0 (rarely or never) to 2 (often) (24). The total loneliness score was obtained by summing the scores of the three items and ranged from 0 to 6, with higher scores indicating more loneliness.

##### 2.4.3.2 Social relations

Social relations were assessed using five non-validate items specifically developed for the COVID-19 cohort study to evaluate participants' feelings of social connectedness and social support. Participants responded to each item on a scale from “totally disagree” to “totally agree” (ranged 0–4). The total score for social relations was obtained by summing the scores from five items, ranging from 0 to 20, with higher scores indicating better social relationships.

### 2.5 Statistical analysis

#### 2.5.1 Statistical methods

Participants were included in this study if they had completed the COVQ at least once and were classified as having moderate-to-severe AD or as without AD. Given the different number of completed COVQs by each participant during each COVID-19 wave, only one observation per participant was included in the analysis. For continuous outcomes, this was achieved by calculating the mean of each participant's outcomes within the same wave. Regarding dichotomous outcomes, current MDD and GAD were diagnosed based on participants reported symptoms at least once across each COVID-19 wave. Continuous outcomes were summarized using means and standard deviations (SDs), and categorical outcomes were described using numbers and proportions.

Generalized linear mixed models (GLMMs) were used to investigate the impact of COVID-19 waves on all the participants. Three GLMMs were performed sequentially for each outcome. Model 1 included COVID-19 waves and AD status, Model 2 was additionally adjusted for sex and age, and Model 3 further included education level, income, and history of mental health problems. Moreover, to investigate whether the impact of COVID-19 waves differed between participants with moderate-to-severe AD and those without AD, an interaction term between COVID-19 waves and AD status was added to the GLMMs. The interaction term was retained in the models if it was statistically significant; otherwise, it was omitted. Results were shown as odds ratio (OR) and 95% confidence interval (CI) for categorical outcomes, estimate and standard error (SE) for continuous outcomes.

In addition, given the availability of data on MDD and GAD history, additional analyses were performed to compare the prevalence of MDD and GAD between the pandemic waves and pre-pandemic period. Population-level differences in MDD prevalence across pandemic waves were compared using Chi-square tests. Pairwise comparisons between the pre-pandemic baseline and each pandemic wave were performed using pairwise proportion tests with Bonferroni correction for multiple testing.

Subsequently, considering the importance of sex in mental health outcomes (25), differences among males without AD, females without AD, males with moderate-to-severe AD, and females with moderate-to-severe AD were further examined. Chi-square tests and Kruskal-Wallis tests were used to compare categorical and continuous outcomes, respectively.

Lastly, although the current study primarily focused on participants with moderate-to-severe AD and those without AD, the impact of the COVID-19 pandemic on individuals with self-reported mild AD was examined in additional GLMM models to explore whether the effects differed by AD severity, applying the same analytical approach as the primary model.

#### 2.5.2 Missing data

Imputation was performed because complete data were required for diagnosing current MDD and GAD and for calculating total scores of loneliness and social relations. The proportion of complete data was assessed prior to imputation (Supplementary Table S3). Assuming data were missing at random, multiple imputation by chained equation (MICE) was applied. Missing data were imputed only if: (1) participants had partially answered questions within a specific COVQ, or (2) certain items were omitted from specific COVQs due to the design of the Lifelines COVID-19 study. Completely missing data, where participants had not responded to any items related to the relevant outcomes, were not imputed.

#### 2.5.3 Non-responder analysis

To evaluate the robustness of the results, non-responder analysis was performed by comparing the study population with COVQ non-responders, defined as those who did not respond to any of the COVQs among the entire Lifelines adult participant population. As the non-responders did not provide any data for the COVQ questions, information on age, sex, body mass index, and education level was extracted from the third Lifelines assessment (2019–2023), which corresponds to a time period comparable to the COVID-19 cohort study, to enable group comparisons. Information on income, smoking, alcohol consumption, and the history of MDD and GAD was obtained from the first two Lifelines assessments, and the most recent available data were used in the current study.

Depending on the variable type, effect sizes were calculated to quantify the magnitude of group differences: Cohen's d for continuous variables, Cohen's h for binary variables, and Cramer's V for multi-level categorical variables. The interpretation of effect sizes followed conventional thresholds proposed by Cohen, where values of ~0.2, 0.5, and 0.8 for Cohen's d (or h), and 0.1, 0.3, and 0.5 for Cramer's V, indicate small, medium, and large effects, respectively (26).

#### 2.5.4 Software

All the statistical analyses were conducted using RStudio (Version 4.4.0) and IBM SPSS Statistics for Windows (Version 28.0, SPSS Inc. Chicago, IL, U.S.A.). Data imputation was performed by mice package in RStudio. *P*-values < 0.05 were considered as statistically significant (two-tailed), and *P*-values were adjusted for multiple comparisons using the Bonferroni method.

## 3 Results

In total, 49,216 participants (including 2,139 with moderate-to-severe AD and 47,077 without AD) completed 790,936 COVQs across 31 time points, which were included in the current analysis (Supplementary Figure S2). Overall, compared with participants without AD, those with moderate-to-severe AD were younger, more often female, and more likely to have a history of MDD or GAD, and had a lower rate of COVID-19 vaccination (all *P* < 0.05, Table 1).

**Table 1:** Description of study population.

**Variables**	**Overall study population**	**Participants without AD**	**Participants with moderate-to-severe AD**	***P-*value**
*N* (%)	49,216	47,077 (95.7)	2,139 (4.3)	
Amount of COVQs	790,936	759,797	31,139	
15.6-2.2,-1.3498ptAge, years, mean ± SD	56.2 ± 12.3	55.5 ± 12.5	51.4 ± 12.9	**< 0.001**
**Age group, years (** ***N*** **, %)**				
18–29	1,585 (3.2)	1,465 (3.1)	120 (5.6)	**< 0.001**
30–44	7,546 (15.3)	7,046 (15.0)	500 (23.4)	
45–59	22,300 (45.3)	21,325 (45.3)	975 (45.6)	
15.6-2.2,-1.3498pt≥60	17,785 (36.1)	17,241 (36.6)	544 (25.4)	
**Sex (** ***N*** **, %)**				
Male	19,737 (40.1)	18,998 (40.4)	739 (34.5)	**< 0.001**
15.6-2.2,-1.3498ptFemale	29,479 (59.9)	28,079 (59.6)	1,400 (65.5)	
**Employment status (** ***N*** **, %)** ^a^				
Student	349 (0.7)	324 (0.7)	25 (1.2)	**< 0.001**
Employed	30,293 (61.6)	28,903 (61.4)	1,390 (65.0)	
Unemployed	1,286 (2.6)	1,205 (2.6)	81 (3.8)	
Retired	11,378 (23.1)	11,069 (23.5)	309 (14.4)	
15.6-2.2,-1.3498ptOthers	5,910 (12.0)	5,576 (11.8)	334 (15.6)	
**Education level (** ***N*** **, %)** ^b^				
Low	9,535 (21.0)	9,220 (21.2)	315 (16.3)	**< 0.001**
Intermediate	17,571 (38.6)	16,810 (38.6)	761 (39.3)	
High	18,367 (40.4)	17,505 (40.2)	862 (44.5)	
15.6-2.2,-1.3498ptMissing	3,743	3,542	201	
**Income level (** ***N*** **, %)**				
Low ( ≤ €1,000)	4,599 (17.7)	4,420 (17.7)	179 (16.8)	0.3
Intermediate (€1,001–€3,000)	18,077 (69.5)	17,316 (69.5)	761 (71.6)	
High (> €3,000)	3,316 (12.8)	3,193 (12.8)	123 (11.6)	
15.6-2.2,-1.3498ptMissing	23,224	22,148	1,076	
**History of MDD (** ***N*** **, %)**				
No	47,176 (97.9)	45,169 (97.9)	2,007 (96.5)	**< 0.001**
Yes	1,029 (2.1)	957 (2.1)	72 (3.5)	
15.6-2.2,-1.3498ptMissing	1,011	951	60	
**History of GAD (** ***N*** **, %)**				
No	45,965 (95.4)	44,041 (95.5)	1,924 (92.5)	**< 0.001**
Yes	2,240 (4.6)	2,085 (4.5)	155 (7.5)	
15.6-2.2,-1.3498ptMissing	1,011	951	60	
**History of mental health problems (** ***N*** **, %)** ^c^				
No	45,541 (94.5)	43,645 (94.6)	1,896 (91.2)	**< 0.001**
Yes	2,664 (5.5)	2,481 (5.4)	183 (8.8)	
15.6-2.2,-1.3498ptMissing	1,011	951	60	
**COVID-19 vaccination**				
No	4,355 (12.8)	4,153 (12.7)	202 (14.8)	**0.026**
Yes	29,631 (87.2)	28,467 (87.3)	1,164 (85.2)	
Missing	15,230	14,457	773	

AD, atopic dermatitis; N, number; COVQ, COVID-19 questionnaires; SD, standard deviation; MDD, major depression disorder; GAD, generalized anxiety disorder.

^a^Employment status refers to the status when participants first filled out the COVQ.

^b^Education level: low (no education, primary education, lower or preparatory secondary vocational education, junior general secondary education), intermediate (secondary vocational education or work-based learning pathway, senior general secondary education, pre-university secondary education), and high (higher vocational education, university education).

^c^History of mental health problems: defined as a history of MDD or GAD.

Bold *P*-values indicate statistical significance (*P* < 0.05).

**Figure 1:**
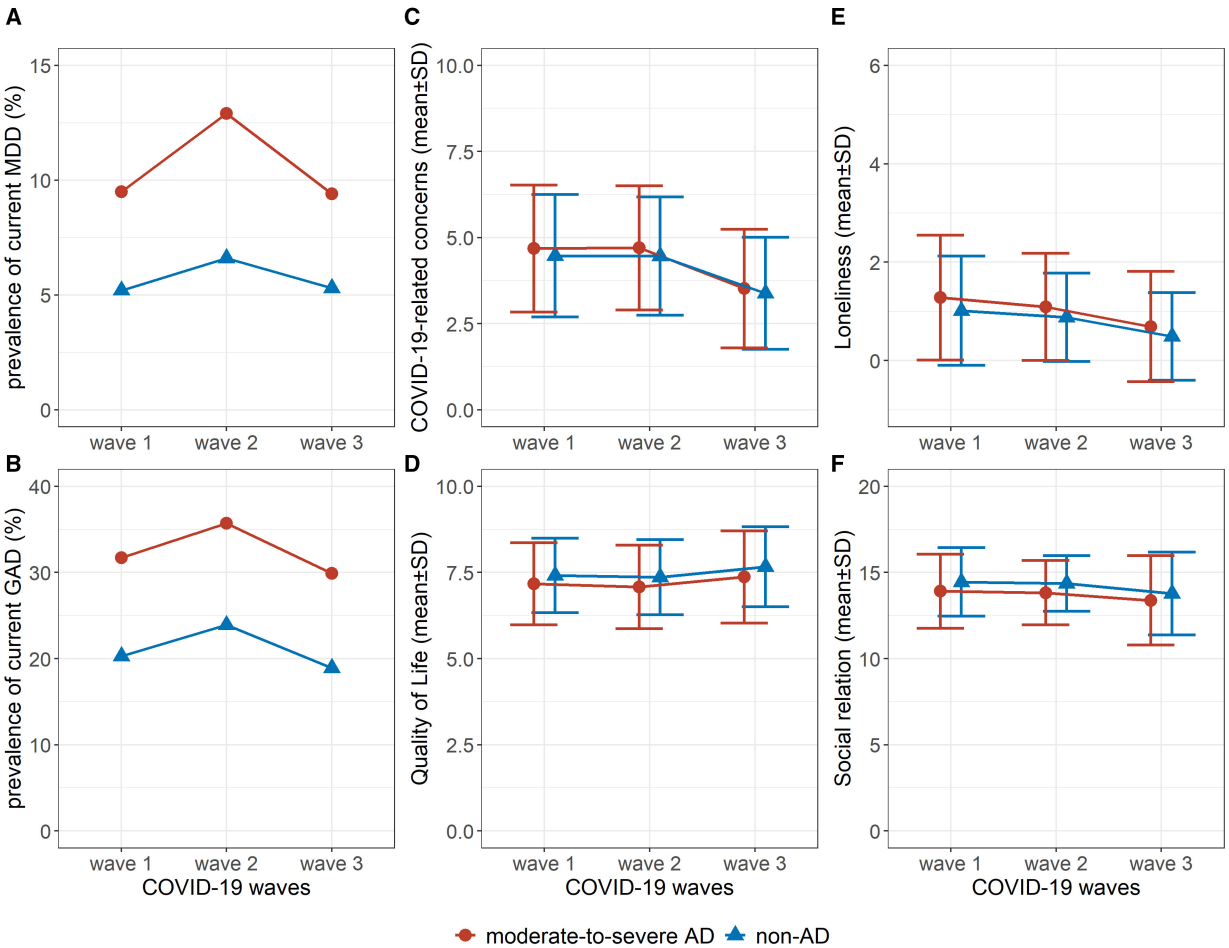
Trajectories of mental health, pandemic-related wellbeing, and social functioning in participants with moderate-to-severe atopic dermatitis (AD) and without AD during the COVID-19 waves. **(A)**. Prevalence of current MDD; **(B)**. Prevalence of current GAD; **(C)**. Score of COVID-19-related concerns; **(D)**. Score of quality of life; **(E)**. Score of loneliness; **(F)**. Score of social relation. COVID-19 waves: wave 1: March 2020—June 2020 (COVQ 1–9), wave 2: July 2020—June 2021 (COVQ 10–21), wave 3: July 2021—October 2022 (COVQ 22–29). AD, atopic dermatitis; MDD, major depressive disorder; GAD, generalized anxiety disorder; COVQ, COVID-19 related questionnaire; SD, standard deviation.

Trajectories of mental health status, pandemic-related wellbeing, and social functioning across the COVID-19 waves among participants with moderate-to-severe AD and those without AD were shown in Figure 1. Compared with participants without AD, those with moderate-to-severe AD showed a higher prevalence of current MDD and GAD, higher COVID-19-related concerns, lower QoL scores, higher loneliness scores, and lower social relations scores across all COVID-19 waves.

The impact of COVID-19 waves on participants with moderate-to-severe AD and those without AD was further evaluated using GLMMs (Table 2). Overall, the patterns were largely consistent across the three models. Adjustment for sociodemographic and mental health history (Model 3) slightly attenuated the associations but did not materially change the direction of the results.

**Table 2:** Effects of COVID-19 waves among participants with moderate-to-severe atopic dermatitis (AD) and those without AD.

**Variables**	**Variable levels**	**Mental health**		**Pandemic-related wellbeing**		**Social functioning**	
		**Current MDD** ^a^	**Current GAD** ^a^	**COVID-19-related concerns**	**QoL**	**Loneliness**	**Social relations**
		**OR (95% CI)**	**OR (95% CI)**	**Estimate (SE)**	**Estimate (SE)**	**Estimate (SE)**	**Estimate (SE)**
**Model 1**							
Intercept		0^***^	0.05 (0.04, 0.05)^***^	4.47 (0.01)^***^	7.41 (0.01)^***^	1.01 (0.005)^***^	14.44 (0.01)^***^
COVID-19 waves^b^	W2 (vs. W1)	1.92 (1.74, 2.11)^***^	1.43 (1.37, 1.50)^***^	−0.004 (0.01)	−0.06 (0.01)^***^	−0.11 (0.004)^***^	−0.10 (0.01)^***^
	W3 (vs. W1)	1.01 (0.91, 1.12)	0.84 (0.80, 0.88)^***^	−1.10 (0.01)^***^	0.24 (0.01)^***^	−0.50 (0.005)^***^	−0.68 (0.01)^***^
AD status	Moderate-to-severe AD (vs. without AD)	2.32 (1.55, 3.48)^***^	4.00 (3.21, 5.00)^***^	0.24 (0.04)^***^	−0.27 (0.03)^***^	0.35 (0.03)^***^	−0.71 (0.05)^***^
COVID-19 waves × AD status	W2, moderate-to-severe AD (vs. W1, without AD)	/	/	/	−0.05 (0.03)	−0.11 (0.03)^***^	/
	W3, moderate-to-severe AD (vs. W1, without AD)	/	/	/	−0.10 (0.03)^**^	−0.13 (0.03)^***^	/
**Model 2**							
Intercept		0^***^	0.09 (0.07, 0.10)^***^	3.87 (0.04)^***^	7.26 (0.03)^***^	1.12 (0.02)^***^	13.33 (0.04)^***^
COVID-19 waves	W2 (vs. W1)	1.92 (1.75, 2.12)^***^	1.46 (1.39, 1.52)^***^	−0.01 (0.01)	−0.06 (0.004)^***^	−0.11 (0.004)^***^	−0.12 (0.01)^***^
	W3 (vs. W1)	1.02 (0.92, 1.14)	0.87 (0.83, 0.91)^***^	−1.10 (0.01)^***^	0.24 (0.01)^***^	−0.50 (0.005)^***^	−0.72 (0.01)^***^
AD status	Moderate-to-severe AD (vs. without AD)	2.04 (1.36, 3.06)^***^	2.77 (2.26, 3.40)^***^	0.22 (0.04)^***^	−0.22 (0.03)^***^	0.30 (0.03)^***^	−0.58 (0.04)^***^
Sex	Female (vs. male)	1.53 (1.31, 1.79)^***^	2.54 (2.38, 2.71)^***^	0.48 (0.01)^***^	−0.14 (0.01)^***^	0.26 (0.01)^***^	0.16 (0.01)^***^
Age group, years	30–44 (vs. 18–29)	0.71 (0.47, 1.06)	0.87 (0.71, 1.05)	0.15 (0.04)^***^	0.13 (0.03)^***^	−0.18 (0.03)^***^	0.47 (0.04)^***^
	45–59 (vs. 18–29)	0.55 (0.38, 0.80)^**^	0.44 (0.37, 0.53)^***^	0.28 (0.04)^***^	0.22 (0.03)^***^	−0.31 (0.02)^***^	0.91 (0.04)^***^
	≥ 60 (vs. 18–29)	0.37 (0.25, 0.55)^***^	0.22 (0.18, 0.26)^***^	0.45 (0.04)^***^	0.30 (0.03)^***^	−0.26 (0.02)^***^	1.44 (0.04)^***^
COVID-19 waves × AD status	W2, moderate-to-severe AD (vs. W1, without AD)	/	/	/	−0.05 (0.03)	−0.11 (0.03)^***^	/
	W3, moderate-to-severe AD (vs. W1, without AD)	/	/	/	−0.10 (0.03)^**^	−0.12 (0.03)^***^	/
**Model 3**							
Intercept		0^***^	0.09 (0.06, 0.14)^***^	3.71 (0.08)^***^	7.30 (0.05)^***^	0.91 (0.05)^***^	13.51 (0.08)^***^
COVID-19 waves	W2 (vs. W1)	2.15 (1.89, 2.44)^***^	1.58 (1.49, 1.68)^***^	0.11 (0.01)^***^	−0.09 (0.01)^***^	−0.09 (0.005)^***^	−0.11 (0.01)^***^
	W3 (vs. W1)	1.09 (0.96, 1.25)	0.90 (0.84, 0.96)^***^	−1.01 (0.01)^***^	0.21 (0.01)^***^	−0.48 (0.01)^***^	−0.69 (0.01)^***^
AD status	Moderate-to-severe AD (vs. without AD)	2.05 (1.34, 3.13)^**^	2.64 (2.15, 3.26)^***^	0.21 (0.05)^***^	−0.21 (0.03)^***^	0.22 (0.03)^***^	−0.35 (0.04)^***^
Sex	Female (vs. male)	1.44 (1.16, 1.80)^**^	2.46 (2.25, 2.70)^***^	0.45 (0.02)^***^	−0.10 (0.01)^***^	0.21 (0.01)^***^	0.13 (0.02)^***^
Age group, years	30–44 (vs. 18–29)	0.71 (0.34, 1.51)	0.73 (0.50, 1.06)	0.09 (0.08)	0.17 (0.05)^**^	−0.15 (0.05)^**^	0.45 (0.07)^***^
	45–59 (vs. 18–29)	0.55 (0.27, 1.13)	0.35 (0.24, 0.50)^***^	0.24 (0.08)^**^	0.25 (0.05)^***^	−0.20 (0.05)^***^	0.86 (0.07)^***^
	≥ 60 (vs. 18–29)	0.37 (0.18, 0.77)^**^	0.17 (0.11, 0.24)^***^	0.46 (0.08)^***^	0.31 (0.05)^***^	−0.13 (0.05)^**^	1.38 (0.07)^***^
Education level^c^	Intermediate (vs. low)	0.94 (0.70, 1.25)	1.10 (0.97, 1.24)	0.01 (0.03)	0.02 (0.02)	−0.001 (0.01)	−0.10 (0.02)^***^
	High (vs. low)	0.88 (0.65, 1.19)	1.21 (1.07, 1.37)^***^	0.08 (0.03)^**^	0.03 (0.02)	0.04 (0.02)^**^	−0.11 (0.02)^***^
Income level^d^	Intermediate (vs. low)	0.77 (0.56, 1.05)	0.71 (0.62, 0.80)^***^	−0.09 (0.03)^**^	0.12 (0.02)^***^	−0.07 (0.02)^***^	0.11 (0.02)^***^
	High (vs. low)	0.92 (0.76, 1.12)	0.85 (0.79, 0.92)^***^	−0.04 (0.02)^*^	0.04 (0.01)^***^	−0.02 (0.01)^*^	−0.02 (0.01)
History of mental health problems^e^	Yes (vs. no)	9.65 (6.84, 13.60)^***^	24.41 (20.00, 29.81)^***^	0.61 (0.04)^***^	−0.74 (0.03)^***^	0.71 (0.02)^***^	−0.40 (0.04)^***^
COVID-19 waves × AD status	W2, moderate-to-severe AD (vs. W1, without AD)	/	/	/	/	−0.06 (0.02)^*^	/
	W3, moderate-to-severe AD (vs. W1, without AD)	/	/	/	/	−0.06 (0.03)^*^	/

^***^P < 0.001, ^**^P < 0.01, ^*^P < 0.05.

MDD, major depression disorder; GAD, generalized anxiety disorder; QoL, quality of life; OR, odds ratio; CI, confidence interval; SE, standard error.

^a^Current MDD and GAD refer to diagnosis made based on participants reported symptoms at least once across each COVID-19 wave.

^b^COVID-19 waves: wave 1 (W1): March 2020—June 2020 (COVQ 1–9), wave 2 (W2): July 2020—June 2021 (COVQ 10–21), wave 3 (W3): July 2021—October 2022 (COVQ 22–29).

^c^Education level: low (no education, primary education, lower or preparatory secondary vocational education, junior general secondary education), intermediate (secondary vocational education or work-based learning pathway, senior general secondary education, pre-university secondary education), and high (higher vocational education, university education).

^d^Income level: low ( ≤ €1,000), intermediate (€1,001–€3,000), high (> €3,000).

^e^History of mental health problems: defined as a history of MDD or GAD.

Results from Model 3 indicating that, compared with W1, the odds of current MDD were higher during W2 (OR, 95% CI: 2.15, 1.89–2.44, *P* < 0.001), and not significantly different in W3 (1.09, 0.96–1.25). The odds of current GAD were higher in W2 compared with W1 (1.58, 1.49–1.68, *P* < 0.001) and lower in W3 (0.90, 0.84–0.96, *P* < 0.001). COVID-19-related concerns decreased from W1 to W3 [Estimate (SE): −1.01 (0.01)], while QoL improved [0.21 (0.01); all *P* < 0.001]. However, score of social relations remained consistently lower during W2 and W3 compared with W1 [W2: −0.11 (0.01) and W3: −0.69 (0.01), both *P* < 0.001]. It is worth noting that although results from Model 3 indicated that the odds of current MDD and GAD in W3 were not significantly different from, or even slightly lower than, those in W1, the population-level prevalence of mental health problems remained elevated. Specifically, compared with the pre-pandemic period, the prevalence of MDD and GAD was significantly higher across all pandemic waves (all *P* < 0.001, Supplementary Table S4).

In addition, compared with those without AD, participants with moderate-to-severe AD had significantly poorer outcomes. They showed significantly higher odds of current MDD (2.05, 1.34–3.13) and current GAD (2.64, 2.15–3.26), higher levels of COVID-19-related concerns [0.21 (0.05)], lower QoL scores [−0.21 (0.05)], higher loneliness scores [0.22 (0.03)], and lower social relations scores (−0.35 (0.04); all *P* < 0.001).

The interaction term between COVID-19 waves and AD status was significant for loneliness (Wave 3 × AD: Estimate = −0.06, SE = 0.03, *P* < 0.05), indicating that the effect of COVID-19 waves on loneliness differed between participants with moderate-to-severe AD and those without AD. Loneliness decreasing from W1 to W3 in both groups, with the decline being slightly more pronounced among participants with moderate-to-severe AD (−0.54 vs. −0.48). Moreover, the estimated marginal means indicated that the differences between participants with moderate-to-severe AD and those without AD were significant across almost all COVID-19 waves (Supplementary Table S5). Overall, similar trends were observed when participants with mild AD were included in the GLMMs, with the odds for all outcomes increasing with the severity of AD (Supplementary Table S6).

Furthermore, females and participants with a history of mental health problems had higher odds of current MDD and GAD, higher COVID-19-related concerns scores, lower QoL scores, higher loneliness scores, and higher social relations scores, whereas older age and higher income were associated with lower odds of current MDD and GAD, better QoL, and less loneliness (all *P* < 0.05).

Additionally, among males without AD, females without AD, males with moderate-to-severe AD, and females with moderate-to-severe AD, the latter showed the highest prevalence of current MDD and GAD, the highest COVID-19-related concerns scores, the lowest QoL scores, and the highest loneliness scores across all COVID-19 waves (Supplementary Table S7). In contrast, males without AD showed the opposite results.

Lastly, the non-responder analysis showed that, compared with the study population, non-responders were younger, included a higher proportion of males, had a higher proportion of individuals with low to intermediate education, low income, higher pack-years of smoking, and a higher likelihood of having a history of MDD or GAD (Supplementary Table S8). Although most characteristics differed significantly between groups, likely due to the large sample size, most effect sizes were small, expect age and history of mental health problems.

## 4 Discussion

In this population-based longitudinal study, the impact of COVID-19 waves on the mental health, pandemic-related wellbeing, and social functioning of participants with moderate-to-severe AD and those without AD in the Dutch general population was investigated. In general, compared with the first COVID-19 wave, most health-related outcomes, including mental health, pandemic-related wellbeing, and QoL, had worsened but returned to levels comparable to those in the first wave by the third wave. However, social relations remained significantly impaired across all waves. Nevertheless, despite this recovery relative to the first wave, mental health levels remained worse than pre-pandemic levels. Additionally, participants with moderate-to-severe AD, women, young adults (18–29 years), and those with a history of mental health problems reported worse health-related outcomes than their counterparts during the COVID-19 pandemic.

Previous studies examining the impact of multiple COVID-19 waves have been limited and have yielded conflicting conclusions. A meta-analysis including diverse populations reported a slight increase in mental health problems after the outbreak, followed by a gradual return to pre-pandemic levels (27). However, the results of the current study indicate that, although the prevalence of mental health problems had returned to levels comparable to those during the first wave of the pandemic, they remained substantially higher than pre-pandemic levels. In addition to the persistent psychological impact associated with the COVID-19 pandemic, the elevated prevalence may partly result from increased detection due to repeated symptom assessments during the pandemic. Furthermore, longitudinal studies from several European countries have shown decreases in depression, anxiety, COVID-19-related concerns, and feelings of social isolation throughout the COVID-19 pandemic (28–30). In contrast, longitudinal studies conducted in Switzerland and Slovenia found increases in depression, anxiety, and loneliness in general population during COVID-19 pandemic (31, 32). These conflicting findings may be explained by differences in follow-up duration and variations in the definition of COVID-19 waves.

Furthermore, as widely reported in previous studies, the QoL of AD patients is significantly affected by intense itching, sleep disturbances, decreased daytime work productivity, and financial burdens, and it often worsens with increasing disease severity (4). Moreover, patients with AD often experience recurring symptoms that require long-term therapy, and switching between different medications is common when treatment is insufficiently effective, especially among those with moderate-to-severe AD (33). As a result, these patients often require in-person dermatology appointments to discuss disease control. In addition, potential psychiatric treatment may be necessary due to sleep deprivation and social avoidance caused by AD. Since lockdown restrictions made access to healthcare more difficult, patients with AD may have faced greater challenges than those without this condition. Furthermore, although few studies have examined the trajectories of health outcomes during the COVID-19 pandemic among individuals with chronic diseases, and comparative studies involving AD are also limited, previous research has reported that individuals with chronic diseases, identified as a vulnerable group, experienced moderate-to-high levels of COVID-19-related fear (34). Consistently, a previous study examining medication-related problems among individuals with chronic diseases during the pandemic found that more than half of the respondents reported a worsening of their conditions, and that those with multiple chronic diseases, psychiatric disorders, or heart failure experienced a significantly higher risk of medical-related problems (35).

In line with this evidence, our study indicated that participants with moderate-to-severe AD had a significantly poorer mental health status during the COVID-19 than those without AD. This finding aligns with a systemic review of eight studies, which reported increased stress, depression, and anxiety among individual with AD, particularly those with moderate-to-severe AD (8, 9, 36–42). A multicenter cross-sectional study of 218 AD patients also highlighted increased discomfort, worries, and social difficulties during the pandemic compared with pre-pandemic levels (10). Similarly, a Polish study of 195 AD patients found that 63% believed individuals with skin diseases were more susceptible to COVID-19 (41), consistent with our findings of heightened pandemic-related concerns among participants with moderate-to-severe AD. Socially, these participants also reported greater loneliness, in line with a Dutch population-based Lifelines study indicating increased loneliness among those with self-reported physician-diagnosed AD, especially moderate-to-severe AD (7). Additionally, an online survey of 279 AD patients across 26 countries found that 43% avoided social situations and 41% preferred solitude to manage their AD-related mental health (43), which may explain poorer self-perceived social relations observed among moderate-to-severe AD participants in our study.

Interestingly, our findings revealed an improvement in loneliness but a concurrent decline in social relations across the COVID-19 waves. This pattern suggests that although individuals reported feeling less emotionally lonely, their social networks and the perceived quality of social relationships may have continued to deteriorate. This finding is consistent with previous studies indicating that emotional recovery does not necessarily correspond to a full restoration of social connectedness (44). This may be explained by the persistently limited opportunities for in-person interactions during the pandemic, such as social gatherings and workplace contacts. Future research should further investigate the underlying reasons for this discrepancy between emotional recovery and social connectedness.

In addition, our study indicated that women, young adults, and individuals with a history of mental health problems had worse mental health status, more impaired pandemic-related wellbeing, and poorer social functioning compared with men, older adults, and those without such a history. These findings are consistent with previous studies reporting that women, younger adults, and individuals with pre-existing mental health problems were more vulnerable than their counterparts during the COVID-19 pandemic (45, 46). This phenomenon may be partly explained by the higher social and medical needs of these groups, which made them more susceptible to the adverse effects of strict lockdown policies.

This study has several strengths. First, it is a large, population-based longitudinal study that included repeated measurements of mental health, pandemic-related wellbeing, and social functioning across 31 time points over more than 2 years. This design provides a more comprehensive understanding of the long-term impact of COVID-19 on the Dutch population. Second, this study contributes to the evidence on the effects of multiple COVID-19 waves by comparing participants with moderate-to-severe AD and those without AD. Third, the scales used to assess MDD, GAD, QoL, and loneliness were validated measures, ensuring the reliability of the findings.

This study has several limitations. First, the questions used to assess COVID-19-related concerns and social relations were not validated, however, they provide valuable insight into the level of concern about the COVID-19 pandemic and social functioning within the population. Second, selection bias may exist because the Lifelines includes only residents from the north of the Netherlands, and participants who did not complete the first seven COVQs were not invited to subsequent follow-ups. The representativeness of the Lifelines Cohort Study has been evaluated by Klijs et al. (47), who reported that the adult Lifelines population is largely representative of adults in the north of the Netherlands, suggesting a low risk of selection bias for this population (47). However, the generalizability of these findings to the national or globally population requires further investigation. Moreover, it is also possible that participants experiencing negative emotions, mental health issues, or social isolation were less likely to complete the COVQs, leading to their underrepresentation. This was supported by the non-response analysis, which showed that non-responders were more likely to have mental health problems than the current study population. Although the effect sizes of these differences were small, our findings might therefore be relatively conservative, and the true levels of mental health problems and wellbeing in the broader populations may have been underestimated. Finally, several potentially relevant factors, such as comorbidities, medication use, and vaccination status were not included in the models in this study due to limitations in data availability and complexity. Individuals with chronic conditions or those who were unvaccinated may have been more vulnerable to the psychological impact of the COVID-19 pandemic. Concerns about infection risk, vaccine safety, and disruptions in routine medical care could have contributed to increased stress, anxiety, and reduced overall wellbeing, while limited access to healthcare services and social support might have further intensified feelings of uncertainty or isolation. Future studies should further investigate these factors to better understand their potential impact.

## 5 Conclusion

In summary, compared with the first COVID-19 wave, both participants with moderate-to-severe AD and those without AD showed slightly improved mental health, fewer COVID-19-related concerns, better QoL, and reduced loneliness by the third wave. However, mental health remained worse than pre-pandemic levels, and social relations continued to be impaired. Participants with moderate-to-severe AD were more affected overall, exhibiting worse mental health, more impaired pandemic-related wellbeing, and poorer social functioning. In addition, women, young adults, and individuals with a history of mental health problems were also more vulnerable to the pandemic's impact than their counterparts.

Future coping strategies should aim to strengthen social relations across the entire population, with particular attention to individuals with moderate-to-severe AD, women, young adults, and those with mental health problems. Strategies such as expanding remote care support, establishing social support groups, creating forums for skin conditions, providing psychological counseling services, and organizing educational campaigns to enhance public awareness of AD may be beneficial. Furthermore, the long-term health impacts of COVID-19 on individuals with and without moderate-to-severe AD require continued attention. In addition, the health status of individuals who are less willing to participate in questionnaires, possibly due to existing mental health issues, should be carefully monitored.

## Data Availability

The data analyzed in this study is subject to the following licenses/restrictions: the data presented in this study are available on request from the Lifelines Biobank. The data are not publicly available due to privacy and ethical reasons. Requests to access these datasets should be directed to Lifelines Biobank: https://www.lifelines.nl.
