# Supplementary material for: Impact of the COVID-19 pandemic waves on adults with moderate-to-severe atopic dermatitis in the Dutch general population: a population-based longitudinal cohort study
Source: Front Public Health. 2025 Dec 2;13:1641763. doi: 10.3389/fpubh.2025.1641763 (PMC12705386; doi:10.3389/fpubh.2025.1641763)
Supplement: Supplementary file 1 [file Table_1.docx]

Supplementary Material

# Supplementary Tables

**Table SI. Overview of items included in the COVID-19 questionnaires**

| **Items** | **No.** | **COVID-19 questionnaires** | | | | | | | | | | | | | | | | | | | | | | | | | | | | | | |
| --- | --- | --- | --- | --- | --- | --- | --- | --- | --- | --- | --- | --- | --- | --- | --- | --- | --- | --- | --- | --- | --- | --- | --- | --- | --- | --- | --- | --- | --- | --- | --- | --- |
|  |  | 1 | 2 | 3 | 4 | 5 | 6 | 7 | 8 | 9 | 10 | 11 | 12 | 13 | 14 | 15 | 15b | 16 | 16b | 17 | 18 | 19 | 20 | 21 | 22 | 23 | 24 | 25 | 26 | 27 | 28 | 29 |
| MDD | A1 |  |  |  |  |  |  |  |  |  |  |  |  |  |  |  |  |  |  |  |  |  |  |  |  |  |  |  |  |  |  |  |
|  | A2 |  |  |  |  |  |  |  |  |  |  |  |  |  |  |  |  |  |  |  |  |  |  |  |  |  |  |  |  |  |  |  |
|  | A3a |  |  |  |  |  |  |  |  |  |  |  |  |  |  |  |  |  |  |  |  |  |  |  |  |  |  |  |  |  |  |  |
|  | A3b |  |  |  |  |  |  |  |  |  |  |  |  |  |  |  |  |  |  |  |  |  |  |  |  |  |  |  |  |  |  |  |
|  | A3c |  |  |  |  |  |  |  |  |  |  |  |  |  |  |  |  |  |  |  |  |  |  |  |  |  |  |  |  |  |  |  |
|  | A3d |  |  |  |  |  |  |  |  |  |  |  |  |  |  |  |  |  |  |  |  |  |  |  |  |  |  |  |  |  |  |  |
|  | A3e |  |  |  |  |  |  |  |  |  |  |  |  |  |  |  |  |  |  |  |  |  |  |  |  |  |  |  |  |  |  |  |
|  | A3f |  |  |  |  |  |  |  |  |  |  |  |  |  |  |  |  |  |  |  |  |  |  |  |  |  |  |  |  |  |  |  |
|  | A3g |  |  |  |  |  |  |  |  |  |  |  |  |  |  |  |  |  |  |  |  |  |  |  |  |  |  |  |  |  |  |  |
| GAD | O1a |  |  |  |  |  |  |  |  |  |  |  |  |  |  |  |  |  |  |  |  |  |  |  |  |  |  |  |  |  |  |  |
|  | O1b |  |  |  |  |  |  |  |  |  |  |  |  |  |  |  |  |  |  |  |  |  |  |  |  |  |  |  |  |  |  |  |
|  | O2 |  |  |  |  |  |  |  |  |  |  |  |  |  |  |  |  |  |  |  |  |  |  |  |  |  |  |  |  |  |  |  |
|  | O3a |  |  |  |  |  |  |  |  |  |  |  |  |  |  |  |  |  |  |  |  |  |  |  |  |  |  |  |  |  |  |  |
|  | O3b |  |  |  |  |  |  |  |  |  |  |  |  |  |  |  |  |  |  |  |  |  |  |  |  |  |  |  |  |  |  |  |
|  | O3e |  |  |  |  |  |  |  |  |  |  |  |  |  |  |  |  |  |  |  |  |  |  |  |  |  |  |  |  |  |  |  |
| COVID-19-related concerns | - |  |  |  |  |  |  |  |  |  |  |  |  |  |  |  |  |  |  |  |  |  |  |  |  |  |  |  |  |  |  |  |
| QoL | - |  |  |  |  |  |  |  |  |  |  |  |  |  |  |  |  |  |  |  |  |  |  |  |  |  |  |  |  |  |  |  |
| Loneliness | a |  |  |  |  |  |  |  |  |  |  |  |  |  |  |  |  |  |  |  |  |  |  |  |  |  |  |  |  |  |  |  |
|  | b |  |  |  |  |  |  |  |  |  |  |  |  |  |  |  |  |  |  |  |  |  |  |  |  |  |  |  |  |  |  |  |
|  | c |  |  |  |  |  |  |  |  |  |  |  |  |  |  |  |  |  |  |  |  |  |  |  |  |  |  |  |  |  |  |  |
| Social relations | a |  |  |  |  |  |  |  |  |  |  |  |  |  |  |  |  |  |  |  |  |  |  |  |  |  |  |  |  |  |  |  |
|  | b |  |  |  |  |  |  |  |  |  |  |  |  |  |  |  |  |  |  |  |  |  |  |  |  |  |  |  |  |  |  |  |
|  | c |  |  |  |  |  |  |  |  |  |  |  |  |  |  |  |  |  |  |  |  |  |  |  |  |  |  |  |  |  |  |  |
|  | d |  |  |  |  |  |  |  |  |  |  |  |  |  |  |  |  |  |  |  |  |  |  |  |  |  |  |  |  |  |  |  |
|  | e |  |  |  |  |  |  |  |  |  |  |  |  |  |  |  |  |  |  |  |  |  |  |  |  |  |  |  |  |  |  |  |
|  | | <-Send out weekly-> | | | | | | <- Send out bi-weekly or monthly -> | | | | | | | | | | | | | | | | | | | | | | | | |

No.: question number in COVID-19 questionnaires; MDD: major depression disorder; GAD: generalized anxiety disorder; QoL: quality of life.

Grids in blue refers to the question was included in the corresponding COVID-19 questionnaire, grids in grey refers to the question was not included.

**Table SII. Detailed items in COVID-19 questionnaires**

| **Items** | **Items in English*** | **Items in Dutch*** | **Range** | **Answer options** |  |
| --- | --- | --- | --- | --- | --- |
|  |  |  |  |  |  |
| MDD | A1. In the last 7/14 days have you felt low or depressed for much of the day, every day? | A1. Hebt u zich tijdens de afgelopen 7/14 dagen voortdurend somber of depressief gevoeld gedurende het grootste gedeelte van de dag, en dit bijna elke dag? | 0/1 | no/yes |  |
|  | A2. In the last 7/14 days have you had the feeling that you've lost interest in or the will to do things you are normally interested in? | A2. Hebt u tijdens de afgelopen 7/14 dagen voortdurend het gevoel gehad nergens meer zin in te hebben of geen interesse meer te hebben voor dingen die u normaal wel interesseren? | 0/1 | no/yes |  |
|  | A3a. In the last 7/14 days did your appetite change noticeably, or did your weight increase or decrease without this being intended? | A3a. Was uw eetlust merkbaar veranderd, of is uw gewicht toegenomen of afgenomen, zonder dat dit de bedoeling was? (In de afgelopen 7/14 dagen) | 0/1 | no/yes |  |
|  | A3b. In the last 7/14 days have you had problems sleeping almost every night (difficulty falling asleep, waking up in the night or too early in the morning, or actually sleeping too much)? | A3b. Hebt u bijna elke nacht slaapproblemen gehad (moeilijk inslapen, wakker worden tijdens de nacht of te vroeg in de ochtend, of juist teveel slapen)? (in de afgelopen 7/14 dagen) | 0/1 | no/yes |  |
|  | A3c. In the last 7/14 days did you speak or move more slowly than normal? Or did you feel restless, jittery and could barely sit still? Nearly every day? | A3c. Praatte of bewoog u trager dan gewoonlijk, of voelde u zich juist rusteloos, gejaagd en kon u moeilijk stil blijven zitten? Bijna elke dag? (In de afgelopen 7/14 dagen) | 0/1 | no/yes |  |
|  | A3d. In the last 7/14 days did you feel tired or without energy almost every day? | A3d. Voelde u zich bijna elke dag moe of futloos? (In de afgelopen 14 dagen) | 0/1 | no/yes |  |
|  | A3e. In the last 7/14 days did you feel worthless or guilty almost every day? | A3e. Voelde u zich bijna elke dag waardeloos of schuldig? (In de afgelopen 7/14 dagen) | 0/1 | no/yes |  |
|  | A3f. In the last 7/14 days was it difficult to concentrate or make decisions almost every day? | A3f. Kon u zich bijna elke dag moeilijk concentreren of moeilijk beslissingen nemen? (In de afgelopen 7/14 dagen) | 0/1 | no/yes |  |
|  | A3g. In the last 7/14 days have you considered hurting yourself, wished you were dead, or had suicidal thoughts? | A3g. Hebt u overwogen zichzelf iets aan te doen, wenste u dat u dood was, of had u zelfmoordgedachten? (In de afgelopen 7/14 dagen) | 0/1 | no/yes |  |
| GAD | O1a. In the last 7/14 days, have you been worrying excessively and worrying about multiple problems of everyday life, at work, at home, in your immediate environment? | O1a. Hebt u in de afgelopen 7/14 dagen buitensporig gepiekerd en zich zorgen gemaakt over meerdere problemen van het dagelijks leven, op het werk, thuis, in uw naaste omgeving? | 0/1 | no/yes |  |
|  | O1b. Were these worries present almost every day in the last 7/14 days? | O1b. Zijn deze zorgen bijna elke dag aanwezig in de afgelopen 7/14 dagen? | 0/1 | no/yes |  |
|  | O2. In the last 7/14 days did you find it hard to set these worries aside or did they prevent you from concentrating? | O2. Vindt u het moeilijk om deze bezorgdheid in de hand te houden of belemmert dit u om zich te concentreren de afgelopen 7/14 dagen? | 0/1 | no/yes |  |
|  | O3a. In the last 7/14 days did it often happen that / You felt restless, jittery or nervous? | O3a. U zich rusteloos, geladen of zenuwachtig voelde? / Gebeurde het in de afgelopen 7 dagen vaak dat | 0/1 | no/yes |  |
|  | O3b. In the last 7/14 days did it often happen that / You felt tense? | O3b. U zich gespannen voelde? / Gebeurde het in de afgelopen 7/14 dagen vaak dat | 0/1 | no/yes |  |
|  | O3e. In the last 7/14 days did it often happen that / You were particularly irritable? | O3e. U bijzonder prikkelbaar was? / Gebeurde het in de afgelopen 7/14 dagen vaak dat | 0/1 | no/yes |  |
| COVID-19-related concerns | How much have you been concerned about the corona crisis in the past 7/14 days? | Hoeveel zorgen maakte u zich de afgelopen 7/14 dagen over de corona-crisis? | 1-10 | not worried - extremely worried |  |
| QoL | How would you rate your quality of life? | Hoe waardeert u uw kwaliteit van leven over de afgelopen 7 / 14 dagen? | 1-10 | terrible - excellent |  |
| Loneliness | a. How often do you feel excluded? | a. Hoe vaak voelt u zich buitengesloten? | 0-2 | rarely or never - often |  |
|  | b. How often do you feel isolated from others? | b. Hoe vaak voelt u zich afgesloten van anderen? | 0-2 | rarely or never - often |  |
|  | c. How often do you feel alone? | c. Hoe vaak voelt u zich alleen? | 0-2 | rarely or never - often |  |
| Social relations | a. I feel connected to all Dutch people | a. Ik voel me verbonden met alle Nederlanders | 0-4 | totally disagree - totally agree |  |
|  | b. I feel connected to my neighbors, family and/or friends | b. Ik voel me verbonden met mijn buren, familie en/of vrienden | 0-4 | totally disagree - totally agree |  |
|  | c. I get the help and support I need from my neighbors, family and/or friends | c. Ik ontvang de hulp en steun die ik nodig heb van mijn buren, familie en/of vrienden | 0-4 | totally disagree - totally agree |  |
|  | d. I do everything I can to help others who are infected with Covid-19 | d. Ik doe alles wat ik kan om anderen te helpen die besmet zijn met het coronavirus | 0-4 | totally disagree - totally agree |  |
|  | e. I expect that others will do everything they can to help me if I get infected or ill with Covid-19 | e. Ik verwacht dat anderen alles zullen doen om mij te helpen als ik met het coronavirus besmet ben of raak | 0-4 | totally disagree - totally agree |  |

* All the items regarding the status in the last 7/14 days.

MDD: major depression disorder; GAD: generalized anxiety disorder; QoL: quality of life.

MDD and GAD diagnosis: the items on sleep symptoms (A3b), fatigue (A3d), and concentration (A3f) were almost identical between MDD and GAD, these items were asked only once in each COVQ to reduce the burden on the participants.

**Table SIII. Proportion of participants with complete outcome data before imputation**

| **Outcomes** | **Diagnostic Criteria*** | **Participants with 100% complete data, n/N (%)** |
| --- | --- | --- |
| Current MDD | Complete data on 9 questions (from A1 to A3g) | 40647/49216 (82.6) |
| Current GAD | Complete data on 6 questions (from O1a to O3e) | 19316/49216 (39.2) |
| Loneliness | Complete data on 3 questions (from a to c) | 48982/49216 (99.5) |
| Social relations | Complete data on 5 questions (from a to e) | 47874/49216 (97.2) |

* Diagnostic criteria: see detailed diagnostic questions in Table SII.

MDD: major depression disorder; GAD: generalized anxiety disorder; n: participants with 100% complete data; N: study population.

**Table SIV. Population-level comparison of mental health status before and during the COVID-19 pandemic across waves**

| **COVID-19 waves** | **MDD prevalence, N (%)** | **GAD prevalence, N (%)** |
| --- | --- | --- |
| Pre-pandemic period | 1161 (2.2) | 2505 (4.8) |
| Wave 1 | 2894 (5.5) | 11147 (21.3) |
| Wave 2 | 3064 (7.1) | 10850 (25.0) |
| Wave 3 | 2004 (5.6) | 7085 (19.8) |

Compare to pre-pandemic level, all adjusted *P* value <0.001. *P* values were adjusted for multiple comparisons using the Bonferroni method.

COVID-19 waves: pre-pandemic period: between 2007 – 2019 ; wave 1 (W1): March 2020 – June 2020 (COVQ 1-9), wave 2 (W2): July 2020 – June 2021 (COVQ 10-21), wave 3 (W3): July 2021 – October 2022 (COVQ 22-29).

MDD: major depression disorder; GAD: generalized anxiety disorder; N: number.

**Table SV. Estimated marginal means of atopic dermatitis (AD) status in each COVID-19 wave**

|  | Mental health | | Pandemic-related well-being | | Social functioning | |
| --- | --- | --- | --- | --- | --- | --- |
|  | **Current MDD** ^a^ | **Current GAD** ^a^ | **COVID-19-related concerns** | **QoL** | **Loneliness** | **Social relations** |
| *Model 1* | Estimate^$^ (SE) | Estimate (SE) | Estimate (SE) | Estimate (SE) | Estimate (SE) | Estimate (SE) |
| W1 ^b^ (ref.) | -0.99 (0.25) *** | -1.47 (0.13) *** | -0.22 (0.05) *** | 0.27 (0.03) *** | -0.35 (0.03) *** | 0.69 (0.06) *** |
| W2 | -0.88 (0.24) *** | -1.37 (0.14) *** | -0.29 (0.06) *** | 0.31 (0.04) *** | -0.24 (0.03) *** | 0.80 (0.07) *** |
| W3 | -0.41 (0.30) | -1.24 (0.16) *** | -0.19 (0.06) ** | 0.37 (0.04) *** | -0.22 (0.03) *** | 0.62 (0.07) *** |
| *Model 2* | Estimate (SE) | Estimate (SE) | Estimate (SE) | Estimate (SE) | Estimate (SE) | Estimate (SE) |
| W1 (ref.) | -0.86 (0.25) *** | -1.10 (0.12) *** | -0.21 (0.05) *** | 0.22 (0.03) *** | -0.31 (0.03) *** | 0.56 (0.06) *** |
| W2 | -0.76 (0.24) ** | -1.00 (0.13) *** | -0.28 (0.06) *** | 0.27 (0.04) *** | -0.20 (0.03) *** | 0.68 (0.07) *** |
| W3 | -0.28 (0.30) | -0.89 (0.15) *** | -0.18 (0.06) ** | 0.33 (0.04) *** | -0.18 (0.03) *** | 0.50 (0.07) *** |
| *Model 3* | Estimate (SE) | Estimate (SE) | Estimate (SE) | Estimate (SE) | Estimate (SE) | Estimate (SE) |
| W1 (ref.) | -0.76 (0.27) ** | -1.02 (0.13) *** | -0.21 (0.05) *** | 0.19 (0.03) *** | -0.22 (0.03) *** | 0.38 (0.05) *** |
| W2 | -0.85 (0.24) *** | -0.93 (0.13) *** | -0.28 (0.05) *** | 0.21 (0.03) *** | -0.16 (0.03) *** | 0.37 (0.06) *** |
| W3 | -0.31 (0.29) | - 0.95 (0.14) *** | -0.13 (0.05) * | 0.23 (0.03) *** | -0.16 (0.03) *** | 0.30 (0.06) *** |

*** *P*<0.001, ** *P*<0.01.

^$^ Estimate: the difference between participants with moderate-to-severe AD and those without AD.

a Current MDD and GAD refer to diagnosis made based on participants reported symptoms at least once across each COVID-19 waves.

b COVID-19 waves: wave 1 (W1): March 2020 – June 2020 (COVQ 1-9), wave 2 (W2): July 2020 – June 2021 (COVQ 10-21), wave 3 (W3): July 2021 – October 2022 (COVQ 22-29).

MDD: major depressive disorder; GAD: generalized anxiety disorder; QoL: quality of life; SE: standard error.

Model 1: included COVID-19 waves and AD status; Model 2: additionally adjusted for sex and age. Model 3:additionally adjusted for education level, income, and history of mental health problems based on Model 2.

**Table SVI. Effects of COVID-19 waves among participants with mild, moderate-to-severe, and no atopic dermatitis (AD)**

|  |  | Mental health | | Pandemic-related well-being | | Social functioning | |
| --- | --- | --- | --- | --- | --- | --- | --- |
|  |  | **Current MDD** ^a^ | **Current GAD** ^a^ | **COVID-19-related concerns** | **QoL** | **Loneliness** | **Social relations** |
| *Model 1* |  | **OR (95%CI)** | **OR (95%CI)** | **Estimate (SE)** | **Estimate (SE)** | **Estimate (SE)** | **Estimate (SE)** |
| Intercept |  | 0 ^***^ | 0.05 (0.046, 0.054) ^***^ | 4.38 (0.01) ^***^ | 7.47 (0.01) ^***^ | 0.94 (0.01) ^***^ | 14.5 (0.01) ^***^ |
| COVID-19 waves ^b^ | W2 (vs. W1) | 1.88 (1.70, 2.06) ^***^ | 1.42 (1.36, 1.49) ^***^ | -0.11 (0.01) ^***^ | -0.09 (0.01) ^***^ | -0.09 (0.01) ^***^ | -0.11 (0.01) ^***^ |
|  | W3 (vs. W1) | 0.99 (0.89, 1.10) | 0.83 (0.79, 0.87) ^***^ | -1.01 (0.01) ^***^ | 0.22 (0.01) ^***^ | -0.48 (0.01) ^***^ | -0.68 (0.01) ^***^ |
| AD | Mild AD (vs. Without AD) | 1.25 (0.78, 1.99) | 1.49 (1.21, 1.85) ^***^ | 0.07 (0.06) | -0.10 (0.04) ^**^ | 0.12 (0.04) ^**^ | -0.28 (0.07) ^***^ |
|  | Moderate-to-severe AD (vs. Without AD) | 2.04 (1.48, 2.81) ^***^ | 3.26 (2.76, 3.85) ^***^ | 0.22 (0.05) ^***^ | -0.26 (0.03) ^***^ | 0.26 (0.03) ^***^ | -0.5 (0.06) ^***^ |
| COVID-19 waves * AD | W2, mild AD (vs. W1, Without AD) | / | / | / | / | -0.05 (0.03) | -0.03 (0.08) |
|  | W3, mild AD (vs. W1, Without AD) | / | / | / | / | -0.06 (0.03) | 0.22 (0.09) ^*^ |
|  | W2, moderate-to-severe AD (vs. W1, Without AD) | / | / | / | / | -0.06 (0.03) ^*^ | -0.02 (0.07) |
|  | W3, moderate-to-severe AD (vs. W1, Without AD) | / | / | / | / | -0.06 (0.03) ^*^ | 0.09 (0.07) |
| *Model 2* |  | **OR (95%CI)** | **OR (95%CI)** | **Estimate (SE)** | **Estimate (SE)** | **Estimate (SE)** | **Estimate (SE)** |
| Intercept |  | 0 ^***^ | 0.09 (0.07, 0.10) ^***^ | 3.82 (0.08) ^***^ | 7.29 (0.05) ^***^ | 1.00 (0.05) ^***^ | 13.4 (0.07) ^***^ |
| COVID-19 waves | W2 (vs. W1) | 1.88 (1.71, 2.07) ^***^ | 1.45 (1.39, 1.51) ^***^ | 0.11 (0.01) ^***^ | -0.09 (0.01) ^***^ | -0.09 (0.01) ^***^ | -0.11 (0.01) ^***^ |
|  | W3 (vs. W1) | 1.00 (0.90, 1.11) | 0.86 (0.82, 0.90) ^***^ | -1.02 (0.01) ^***^ | 0.21 (0.01) ^***^ | -0.48 (0.01) ^***^ | -0.69 (0.01) ^***^ |
| AD | Mild AD (vs. Without AD) | 1.23 (0.77, 1.95) | 1.43 (1.17, 1.74) ^***^ | 0.13 (0.06) | -0.11 (0.04) ^**^ | 0.14 (0.04) ^***^ | -0.23 (0.07) ^**^ |
|  | Moderate-to-severe AD | 1.87 (1.36, 2.58) ^***^ | 2.52 (2.16, 2.94) ^***^ | 0.23 (0.05) ^***^ | -0.23 (0.03) ^***^ | 0.24 (0.03) ^***^ | -0.40 (0.06) ^***^ |
| Sex | Female (vs. Male) | 1.53 (1.31, 1.79) ^***^ | 2.54 (2.38, 2.71) ^***^ | 0.48 (0.02) ^***^ | -0.14 (0.01) ^***^ | 0.24 (0.01) ^***^ | 0.09 (0.02) ^***^ |
| Age group, yrs. | 30 – 44 (vs. 18-29) | 0.71 (0.48, 1.05) | 0.85 (0.71, 1.03) | 0.08 (0.08) | 0.17 (0.05) ^**^ | -0.17 (0.05) ^***^ | 0.46 (0.07) ^***^ |
|  | 45 – 59 (vs. 18-29) | 0.56 (0.39, 0.80) ^**^ | 0.44 (0.37, 0.53) ^***^ | 0.21 (0.08) ^**^ | 0.25 (0.05) ^***^ | -0.24 (0.05) ^***^ | 0.87 (0.07) ^***^ |
|  | ≥ 60 (vs. 18-29) | 0.38 (0.26, 0.55) ^***^ | 0.22 (0.18, 0.27) ^***^ | 0.42 (0.08) ^***^ | 0.31 (0.05) ^***^ | -0.18 (0.05) ^***^ | 1.41 (0.07) ^***^ |
| COVID-19 waves * AD | W2, mild AD (vs. W1, Without AD) | / | / | / | / | -0.05 (0.03) | -0.03 (0.08) |
|  | W3, mild AD (vs. W1, Without AD) | / | / | / | / | -0.07 (0.03) | 0.22 (0.09) ^*^ |
|  | W2, moderate-to-severe AD (vs. W1, Without AD) | / | / | / | / | -0.06 (0.03) ^*^ | 0.02 (0.07) |
|  | W3, moderate-to-severe AD (vs. W1, Without AD) | / | / | / | / | -0.06 (0.03) ^*^ | 0.08 (0.07) |
| *Model 3* |  | **OR (95%CI)** | **OR (95%CI)** | **Estimate (SE)** | **Estimate (SE)** | **Estimate (SE)** | **Estimate (SE)** |
| Intercept |  | 0 ^***^ | 0.09 (0.06, 0.13) ^***^ | 3.72 (0.08) ^***^ | 7.32 (0.05) ^***^ | 0.92 (0.05) ^***^ | 13.5 (0.07) ^***^ |
| COVID-19 waves | W2 (vs. W1) | 2.12 (1.87, 2.40) ^***^ | 1.57 (1.49, 1.67) ^***^ | 0.11 (0.01) ^***^ | -0.09 (0.01) ^***^ | -0.09 (0.01) ^***^ | -0.11 (0.01) ^***^ |
|  | W3 (vs. W1) | 1.07 (0.94, 1.22) | 0.89 (0.83, 0.95) ^***^ | -1.02 (0.01) ^***^ | 0.21 (0.01) ^***^ | -0.48 (0.01) ^***^ | -0.69 (0.01) ^***^ |
| AD | Mild AD (vs. without AD) | 1.22 (0.66, 2.28) | 1.26 (0.96, 1.65) | 0.11 (0.06) | -0.10 (0.04) ^**^ | 0.13 (0.04) | -0.22 (0.07) ^**^ |
|  | Moderate-to-severe AD (vs. Without AD) | 2.05 (1.35, 3.13) ^***^ | 2.64 (2.15, 3.25) ^***^ | 0.21 (0.05) ^***^ | -0.21 (0.03) ^***^ | 0.22 (0.03) ^***^ | -0.38 (0.06) ^***^ |
| Sex | Female (vs. Male) | 1.44 (1.16, 1.78) ^***^ | 2.45 (2.24, 2.68) ^***^ | 0.45 (0.02) ^***^ | -0.10 (0.01) ^***^ | 0.22 (0.01) ^***^ | 0.13 (0.02) ^***^ |
| Age group, yrs. | 30 – 44 (vs. 18 – 29) | 0.70 (0.33, 1.45) | 0.77 (0.53, 1.10) | 0.10 (0.08) | 0.15 (0.05) ^**^ | -0.15 (0.05) ^**^ | 0.45 (0.07) ^***^ |
|  | 45 – 59 (vs. 18 – 29) | 0.54 (0.27, 1.09) | 0.37 (0.26, 0.52) ^***^ | 0.25 (0.08) ^**^ | 0.23 (0.05) ^***^ | -0.21 (0.05) ^***^ | 0.85 (0.07) ^***^ |
|  | ≥ 60 (vs. 18 – 29) | 0.37 (0.18, 0.74) ^**^ | 0.17 (0.12, 0.25) ^***^ | 0.48 (0.08) ^***^ | 0.30 (0.05) ^***^ | -0.13 (0.05) ^**^ | 1.37 (0.07) ^***^ |
| Education ^c^ | Middle (vs. Low) | 0.93 (0.70, 1.23) | 1.09 (0.97, 1.23) | 0.01 (0.03) | 0.01 (0.02) | 0 (0.02) | -0.10 (0.02) ^***^ |
|  | High (vs. Low) | 0.88 (0.65, 1.18) | 1.20 (1.06, 1.36) ^**^ | 0.08 (0.03) ^**^ | 0.03 (0.02) | 0.05 (0.02) ^**^ | -0.11 (0.02) ^***^ |
| Income ^d^ | Middle (vs. Low) | 0.77 (0.57, 1.05) | 0.72 (0.64, 0.81) ^***^ | -0.09 (0.03) ^***^ | 0.12 (0.02) ^***^ | -0.07 (0.02) ^***^ | 0.11 (0.02) ^***^ |
|  | High (vs. Low) | 0.92 (0.76, 1.11) | 0.86 (0.80, 0.93) ^***^ | -0.04 (0.02) ^*^ | 0.04 (0.01) ^***^ | -0.02 (0.01) ^*^ | -0.02 (0.01) |
| History of mental health problems ^e^ | Yes (vs. No) | 9.82 (7.00, 13.80) ^***^ | 23.6 (19.4, 28.7) ^***^ | 0.62 (0.04) ^***^ | -0.74 (0.03) ^***^ | 0.71 (0.02) ^***^ | -0.40 (0.04) ^***^ |
| COVID-19 waves * AD | W2, mild AD (vs. W1, Without AD) | / | / | / | / | 0.05 (0.03) | -0.40 (0.04) |
|  | W3, mild AD (vs. W1, Without AD) | / | / | / | / | -0.06 (0.03) ^*^ | 0.22 (0.09) ^*^ |
|  | W2, moderate-to-severe AD (vs. W1, Without AD) | / | / | / | / | -0.06 (0.03) ^*^ | 0.02 (0.07) |
|  | W3, moderate-to-severe AD (vs. W1, Without AD) | / | / | / | / | -0.06 (0.03) ^*^ | 0.08 (0.07) |

*** *P* < 0.001, ** *P* < 0.01, * *P* < 0.05.

MDD: major depression disorder; GAD: generalized anxiety disorder; QoL: quality of life; OR: odds ratio; CI: confidence interval; SE: standard error.

**^a^** Current MDD and GAD refer to diagnosis made based on participants reported symptoms at least once across each COVID-19 wave.

**^b^** COVID-19 waves: wave 1 (W1): March 2020 – June 2020 (COVQ 1-9), wave 2 (W2): July 2020 – June 2021 (COVQ 10-21), wave 3 (W3): July 2021 – October 2022 (COVQ 22-29).

**^c^** Education level: low (no education, primary education, lower or preparatory secondary vocational education, junior general secondary education), intermediate (secondary vocational education or work-based learning pathway, senior general secondary education, pre-university secondary education), and high (higher vocational education, university education).

**^d^** Income level: low (≤ €1000), intermediate (€1001 - €3000), high (> €3000).

**^e^** History of mental health problems: defined as a history of MDD or GAD.

**Table SVII. Comparison of mental health, pandemic-related well-being, and social functioning during each COVID-19 wave by status of atopic dermatitis (AD) and sex**

| **Outcomes** | **Range** | **Answer options** | **COVID-19 waves** ^f^ | **Participants without AD** | | **Participants with moderate-to-severe AD** | |
| --- | --- | --- | --- | --- | --- | --- | --- |
|  |  |  |  | Male | Female | Male | Female |
| Current MDD (%) ^e^ | - | - | wave 1 | 3.8 ^a^ | 6.1 ^b^ | 7.4 ^b, c^ | 10.6 ^c^ |
|  |  |  | wave 2 | 5.1 ^a^ | 7.6 ^b^ | 10.6 ^c^ | 14.1 ^c^ |
|  |  |  | wave 3 | 4.3 ^a^ | 6.0 ^b^ | 7.1 ^b, c^ | 10.6 ^c^ |
| Current GAD(%) ^e^ | - | - | wave 1 | 14.6 ^a^ | 24.2 ^b^ | 25.8 ^b^ | 34.7 ^c^ |
|  |  |  | wave 2 | 17.7 ^a^ | 28.0 ^b^ | 26.5 ^b^ | 40.6 ^c^ |
|  |  |  | wave 3 | 14.2 ^a^ | 22.1 ^b^ | 24.0 ^b^ | 33.1 ^c^ |
| COVID-19-related concerns, mean± SD | 1-10 | Higher score, more worry | wave 1 | 4.15 ± 1.80 ^a^ | 4.69 ± 1.73 ^b^ | 4.27 ± 1.91 ^a^ | 4.90 ± 1.77 ^c^ |
|  |  |  | wave 2 | 4.15 ± 1.74 ^a^ | 4.66 ± 1.67 ^b^ | 4.34 ± 1.86 ^c^ | 4.89 ± 1.74 ^d^ |
|  |  |  | wave 3 | 3.22 ± 1.63 ^a^ | 3.49 ± 1.63 ^b^ | 3.31 ± 1.73 ^a^ | 3.63 ± 1.71 ^c^ |
| QoL, mean± SD | 1-10 | Higher score, better QoL | wave 1 | 7.52 ± 1.10 ^a^ | 7.35 ± 1.05 ^b^ | 7.27 ± 1.25 ^b^ | 7.12 ± 1.16 ^c^ |
|  |  |  | wave 2 | 7.46 ± 1.13 ^a^ | 7.30 ± 1.06 ^b^ | 7.11 ± 1.36 ^b^ | 7.06 ± 1.13 ^c^ |
|  |  |  | wave 3 | 7.74 ± 1.20 ^a^ | 7.63 ± 1.14 ^b^ | 7.41 ± 1.42 ^c^ | 7.34 ± 1.30 ^d^ |
| Loneliness, mean± SD | 0-6 | Higher score, more lonely | wave 1 | 0.79 ± 0.99 ^a^ | 1.15 ± 1.16 ^b^ | 1.00 ± 1.19 ^c^ | 1.43 ± 1.29 ^d^ |
|  |  |  | wave 2 | 0.73 ± 0.83 ^a^ | 0.98 ± 0.94 ^b^ | 0.95 ± 1.07 ^c^ | 1.17 ± 1.10 ^d^ |
|  |  |  | wave 3 | 0.41 ± 0.83 ^a^ | 0.54 ± 0.93 ^b^ | 0.56 ± 1.08 ^b^ | 0.75 ± 1.14 ^c^ |
| Social relations, mean± SD | 0-20 | Higher score, better social relations | wave 1 | 14.34 ± 2.08 ^a^ | 14.52 ± 1.92 ^b^ | 13.68 ± 2.25 ^c^ | 14.03 ± 2.09 ^d^ |
|  |  |  | wave 2 | 14.38 ± 1.65 ^a^ | 14.34 ± 1.59 ^b^ | 13.88 ± 2.01 ^c^ | 13.78 ± 1.80 ^d^ |
|  |  |  | wave 3 | 13.76 ± 2.48 ^a^ | 13.79 ± 2.34 ^a^ | 13.31 ± 2.70 ^b^ | 13.42 ± 2.53 ^b^ |

Proportions shared with same superscript letters in each wave indicates they do no differ significantly from each other at the 0.05 level.

^e^ Current MDD and GAD refer to diagnosis made based on participants reported symptoms at least once across each COVID-19 waves.

^f^ COVID-19 waves: wave 1: March 2020 – June 2020 (COVQ 1-9), wave 2: July 2020 – June 2021 (COVQ 10-21), wave 3: July 2021 – October 2022 (COVQ 22-29).

AD: atopic dermatitis; MDD: major depressive disorder; GAD: generalized anxiety disorder; SD: standard deviation; QoL: quality of life; COVQ: COVID-19 related questionnaire.

**Table SVIII. Non-responder analysis: Comparison between the study population and COVQ non-responders**

|  | Overall | COVQ Non-responders* | Study population | *P* value | Effect size (95% CI) |
| --- | --- | --- | --- | --- | --- |
| N (%) | 125628 | 76412 (60.8) | 49216 (39.2) |  |  |
| Age, yrs. mean ± SD | 54.3 ± 13.7 | 49.5 ± 15.3 | 57.1 ± 12.0 | **<0.001** | -0.58 (-0.60, -0.56) |
| Sex, N (%) |  |  |  | **<0.001** | 0.07 (0.06, 0.08) |
| Male | 49846 (42.2) | 30109 (43.6) | 19737 (40.1) |  |  |
| Female | 68364 (57.8) | 38885 (56.4) | 29479 (59.9) |  |  |
| *Missing* | *7418* | *7418* | *0* |  |  |
| Body mass index, mean ± SD | 26.8 ± 4.6 | 26.7 ± 4.7 | 26.8 ± 4.5 | 0.080 | -0.01 (-0.03, 0.00) |
| *Missing* | *63011* | *53433* | *9578* |  |  |
| Education level, N (%) |  |  |  | **<0.001** | 0.12 (0.12, 0.13) |
| Low | 20594 (25.1) | 11059 (30.1) | 9535 (21.0) |  |  |
| Intermediate | 31994 (38.9) | 14423 (39.3) | 17571 (38.6) |  |  |
| High | 29597 (36.0) | 11230 (30.6) | 18367 (40.4) |  |  |
| *Missing* | *43443* | *39700* | *3743* |  |  |
| Income level, N (%) |  |  |  | **<0.001** | 0.11 (0.10, 0.11) |
| Low | 16960 (18.4) | 11204 (21.7) | 5756 (14.1) |  |  |
| Intermediate | 58252 (63.0) | 31841 (61.7) | 26411 (64.7) |  |  |
| High | 17196 (18.6) | 8529 (16.5) | 8677 (21.2) |  |  |
| *Missing* | *33220* | *24838* | *8382* |  |  |
| Smoking, pack-year, mean ± SD | 6.22 ± 10.02 | 6.37 ± 10.31 | 6.04 ± 9.63 | **0.017** | 0.03 (0.02, 0.05) |
| *Missing* | *19411* | *16805* | *2606* |  |  |
| Alcohol consumption, drinks/day, mean ± SD | 0.77 ± 1.00 | 0.77 ± 1.03 | 0.77 ± 0.98 | **<0.001** | 0.00 (-0.02, 0.01) |
| *Missing* | *22687* | *18630* | *4057* |  |  |
| History of MDD, N (%) |  |  |  | **<0.001** | -0.25 (-0.27, -0.24) |
| No | 54012 (90.5) | 22466 (86.3) | 31546 (93.8) |  |  |
| Yes | 5665 (9.5) | 3569 (13.7) | 2096 (6.2) |  |  |
| *Missing* | *65951* | *50377* | *15574* |  |  |
| History of GAD, N (%) |  |  |  | **<0.001** | -0.28 (-0.30, -0.27) |
| No | 50156 (80.6) | 20773 (74.4) | 29383 (85.7) |  |  |
| Yes | 12062 (19.4) | 7143 (25.6) | 4919 (14.3) |  |  |
| *Missing* | *63410* | *48496* | *14914* |  |  |

*COVQ non-responders: defined as those who did not respond to any of the Lifelines COVID-19 study questionnaires (COVQs) among the entire Lifelines adult participant population.

MDD: major depressive disorder; GAD: generalized anxiety disorder; SD: standard deviation; QoL: quality of life; COVQ: COVID-19 related questionnaire; CI: confidence interval.

# Fig S1. Definition of participants with AD and without AD


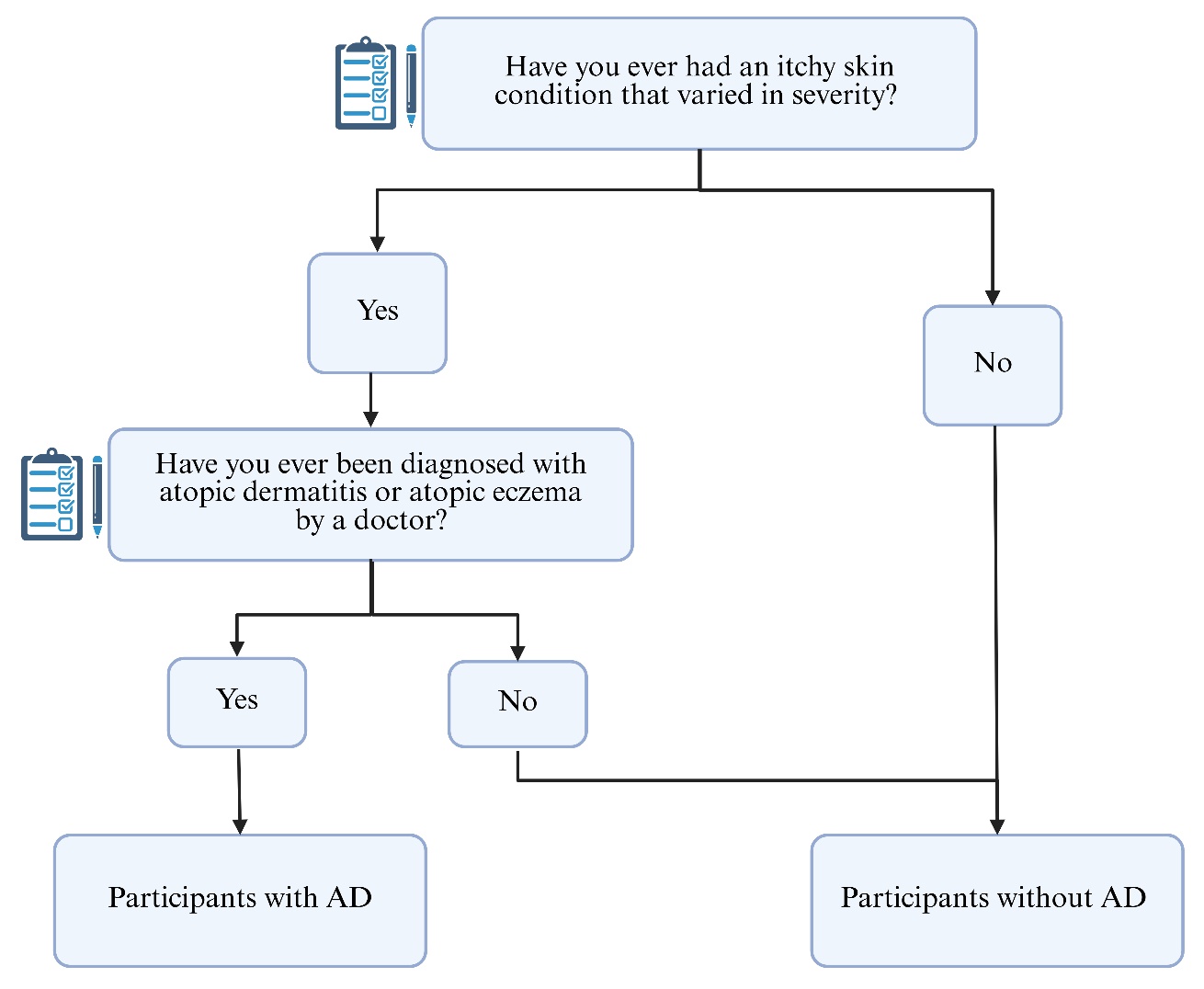


AD: atopic dermatitis.

**Fig S2. Flow diagram of study population selection**


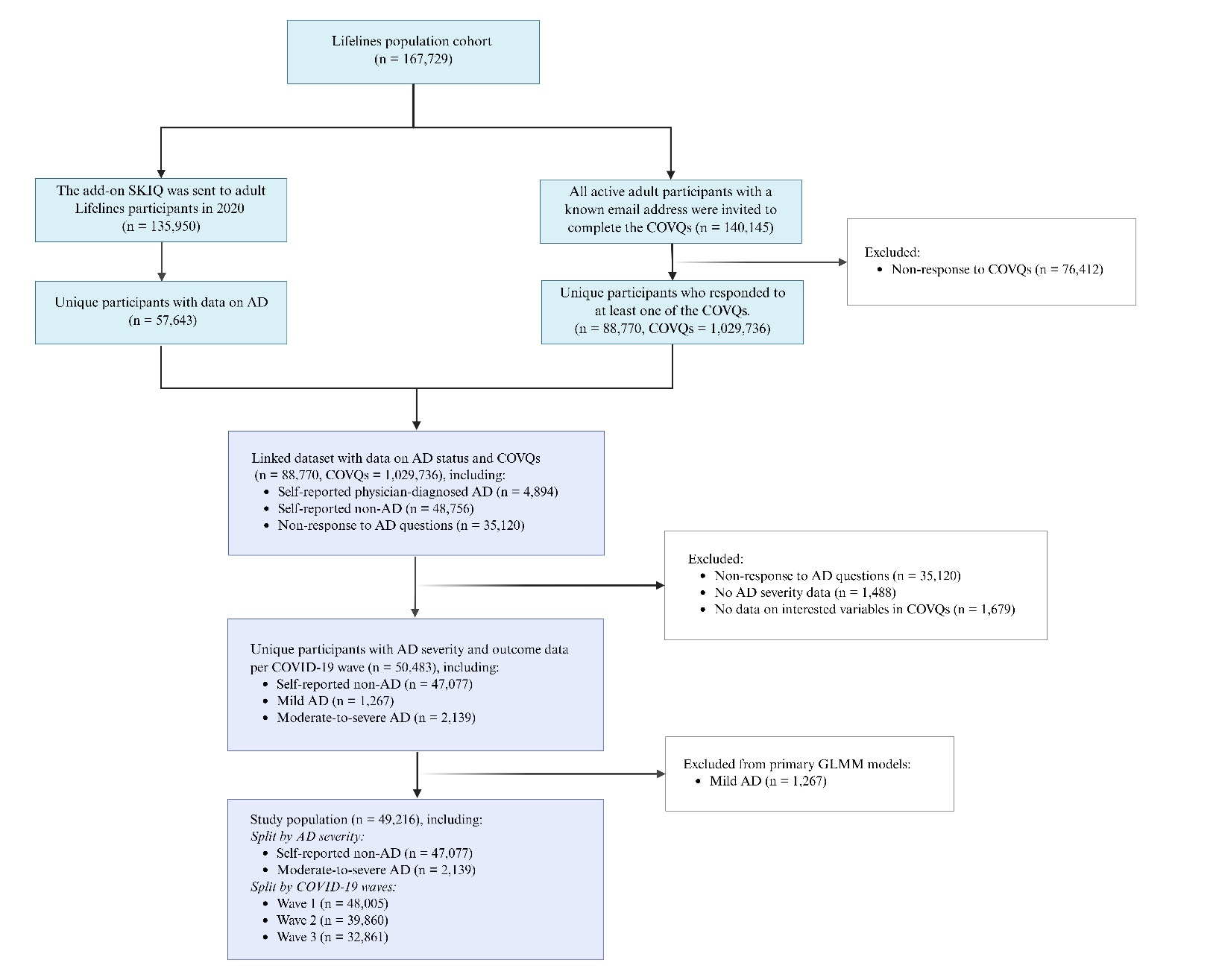


COVQs: COVID-19 questionnaires; SKIQ: skin questionnaire; AD: atopic dermatitis.
